# An annotated image dataset of medically and forensically important flies for deep learning model training

**DOI:** 10.1038/s41597-022-01627-5

**Published:** 2022-08-20

**Authors:** Song-Quan Ong, Hamdan Ahmad

**Affiliations:** 1 Institute for Tropical Biology and Conservation, Universiti Malaysia Sabah, Jalan UMS, 88400, Kota Kinabalu, Sabah Malaysia; 2 Vector Control Research Unit, School of Biological Sciences, Universiti Sains Malaysia, 11800 Penang, Malaysia

**Keywords:** Classification and taxonomy, Entomology

## Abstract

Conventional methods to study insect taxonomy especially forensic and medical dipterous flies are often tedious, time-consuming, labor-intensive, and expensive. An automated recognition system with image processing and computer vision provides an excellent solution to assist the process of insect identification. However, to the best of our knowledge, an image dataset that describes these dipterous flies is not available. Therefore, this paper introduces a new image dataset that is suitable for training and evaluation of a recognition system involved in identifying the forensic and medical importance of dipterous flies. The dataset consists of a total of 2876 images, in the input dimension (224 × 224 pixels) or as an embedded image model (96 × 96 pixels) for microcontrollers. There are three families (Calliphoridae, Sarcophagidae, Rhiniidae) and five genera (Chrysomya, Lucilia, Sarcophaga, Rhiniinae, Stomorhina), and each class of genus contained five different variants (same species) of fly to cover the variation of a species.

## Abstract (summary)

**Table:** 

Measurement(s)	supervised machine learning
Technology Type(s)	Camera Device
Sample Characteristic - Organism	Diptera sp. NZAC 03009335

## Background & Summary

Flies have strongly associated with various microorganisms such as bacteria, viruses, protozoa, fungi, and helminth parasites. Some species are serious medical pests due to their capability as mechanical vectors that carry pathogens or parasitize livestock or humans, causing myiasis^1–3^. Other species called carrion flies are forensically important and considered important scavengers due to their necrophagous feeding behaviors^4^. In terms of forensic entomology, some species provide an alternative way to estimate the minimum post-mortem interval (PMI) of a victim in forensic investigations^5^. Flies are important in many different fields and show great diversity in morphology, behavior, and ecology. Conventional taxonomy and systematic identification of the flies especially those involved in forensic and medical still rely heavily on human observation with or without the aid of microscopic tools. However, these methods are often tedious, time-consuming, labor-intensive, and expensive. Therefore, computer vision and deep learning could provide an excellent alternative for these global challenges, and a suitable dataset is a key to an accurate and reliable machine learning model. This paper provides an image dataset that has three common families of flies that are crucial in medical and forensic entomology. The images were formatted in a JPEG file, processed into 224 × 224 pixels for machine learning or convolutional neural network (CNN) training, and 96 × 96 pixels with smaller file size, as an embedded image model for the microcontroller. Both dimensions consist of 96 dpi resolution and 24-bit depth, and images were annotated into taxonomy levels of genus.

**Fig. 1:**
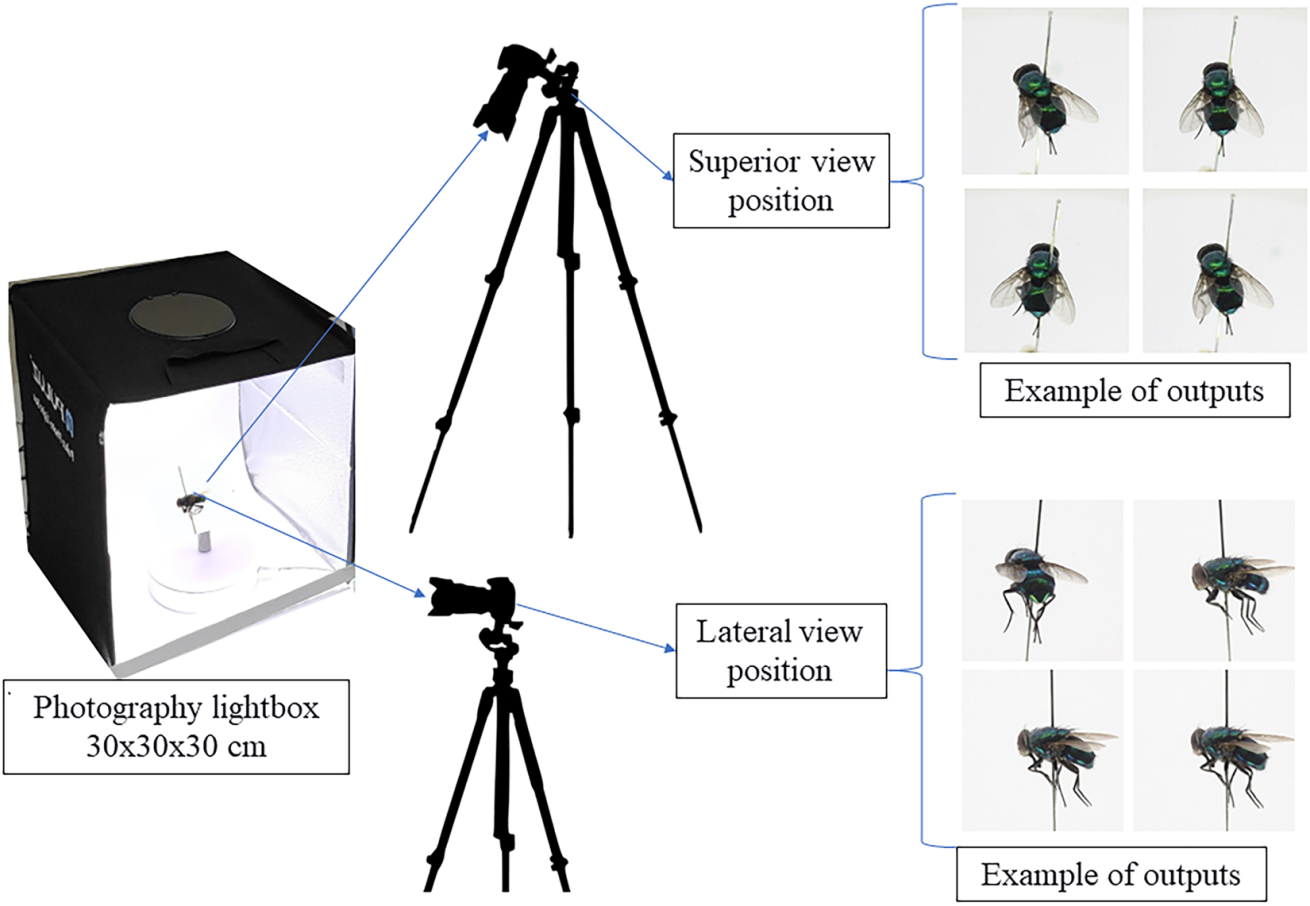
General workflow to record the dataset and organised into the labelled classes.

The image dataset is a raw data that could serve as an authenticated dataset in recognise three families or five genera of medical and forensically important flies. Subsequently, the dataset could be used by potential user such as machine learning engineer, apps developer, data scientist, taxonomist, medical and forensic entomology etc. Figure 1 illustrate the general workflow to record the dataset and organised into the labelled classes, and Table 1 summarizes the structure and labels of the dataset.

**Table 1:** Summary of the image annotations.

Annotation (number of images)	
Family	Genera
• Calliphoridae (1318)	• Chrysomya (731)
• Sarcophagidae (570)	• Lucilia (587)
• Rhiniidae (988)	• Sarcophaga (570)
	• Rhiniinae (488)
	• Stomorhina (500)

## Methods

### Resources of insect specimen

The insect specimens were obtained from the Insect Collection Room of Borneensis, Institute for Tropical Biology and Conservation (ITBC), Universiti Malaysia Sabah (UMS). The Insect Collection Room kept more than 200,000 insect specimens that have been preserved and stored in a compactor at temperature of 18 °C and humidity of 40 ± 5%. The taxonomy of insects has been identified and validated until the taxonomy of genus-level by two taxonomists. The adult stage of the insects were used for image acquisition, and the annotation of the dataset was set on the genus level.

### Data collection

**Fig. 2:**
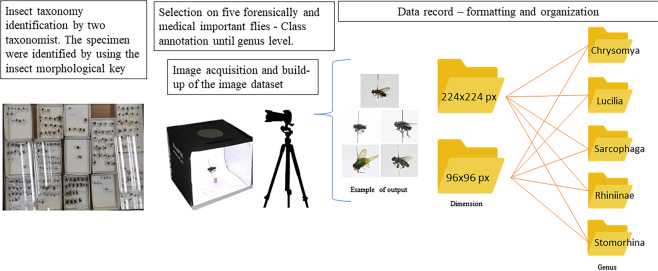
Data collection process.

The insects’ images were acquired by a digital single-lens reflex (DSLR) camera (Canon EOS 50D, 15.0 MP APS-C CMOS Sensor) with Tamron 90 mm f/2.8 Di Macro. The image acquisitions process was conducted in a photography lightbox 30x30x30 cm (Fig. 2) with 34 W white light illumination. The insect specimen was placed in a pin on an electronic motorized rotating plate (the 30 s per resolution) and the camera acquired the images with three-frames per second. The images of the insect were acquired at two levels of position – superior view and lateral view of the insect (Fig. 2). The quality of the images (sharpness, brightness etc.) was checked after the acquisition, poor quality images were removed and caused the genera having different total number of images (Table 1). Table 2 shows the description and example of annotated classes of flies.

**Table 2:** Description and example of annotated classes of flies.

Order	Family	Genus	Examples
Diptera	Calliphoridae	Chrysomya	
		Lucilia	
	Sarcophagidae	Sarcophaga	
	Rhiniidae	Rhiniinae	
		Stomorhina	

### Ethics Statements

All authors confirm that we have complied with all relevant ethical regulations.

## Data Records

The dataset is publicly available in figshare, with direct URL to data: 10.6084/m9.figshare.19607193.v2^6^. Figure 1 illustrated the general workflow to record the dataset and organised it into the labeled classes. In general, after the images were acquired from the museum specimen, they were formatted into 224×224 for DCNN model training or 96×96 for embedded image model training. Users can train the model based on the label of genus – five classes. The image of a genus consists of 5 variants of specimens and consists of 360° view of a specimen. Therefore, for further species level identification by other user (to build a species level recognition system), we re-organized the images according to the individual specimen, and supply as a folder in this dataset.

## Technical Validation

### Taxonomy

The taxonomy of insects has been identified by two taxonomists based on the morphological practical keys to families, subfamilies, and genera as described by^4,7–9^.

### A pilot test with a model build-up

We conducted a pilot test on the datasets to validate the quality in the terms of the development of a deep convolutional neural networks (DCNN) model. We utilize a web-based tool from Google Creative Lab—Teachable Machine 2.0—that is able to train a computational model with no coding required^10^.

The data splitting conducted on this dataset that used for training and testing are: - training (85%) and the prediction is carried out on the testing split (15%), which the images were randomly selected and not repeated with the train split. The platform also allows us to fine-tune the model with hyperparameters, such as the learning rate, batch size, and epoch. For the purpose of dataset quality validation but not presenting a new interpretations of deep learning model construction, this pilot test standardized the batch size to 16 and epoch to 50, and we only fine-tune with three levels of learning rate – 0.0001, 0.001, 0.01 to demonstrate the output of models by using the datasets Table 3 shows the result of the accuracy for the train and test split of the dataset, respectively. The learning curve consists of accuracy on the y-axis, which is the evaluation matric of the probability of accurate prediction against the epoch on the x-axis, which is the number of passes of the entire training dataset the deep learning algorithm has completed)0^11^. Table 4 shows the loss for the train and test split of the dataset, respectively. The function loss curve consists of a loss function on the y-axis, which is a measurement of the differences between predicted and true values against the epoch on the x-axis. Table 5 shows the confusion matrix from the prediction on the test split (based on 433 images), which is a summary of prediction results that consists of correct and incorrect predictions (Prediction against the true value)^11^. or more machine learning model evaluation metrics such as precision and recall can be obtained from the confusion matrix as described in^12^.

**Table 3:** Pilot test result: Training and testing accuracy of the deep learning model by using two different dimensions of dataset at three learning rates; blue line is representing training accuracy; orange line is representing testing accuracy.

**Table 4:** Pilot test result: Training and testing loss of a deep learning model by using two different dimensions of dataset at three learning rates; blue line is representing training function loss, orange line is representing testing function loss .

**Table 5:** Confusion matrix of the deep learning model by using two different dimensions of dataset at three learning rates; the blue intensities indicate the frequency counts, the darker the blue colour the higher the frequency.

Chy- Chrysomya; Luc- Lucilia; Sto- Stomorhina; Sar- Sarcophagidae; Rhi- Rhiniinae

Number of images used for pilot test training and testing [class (train: test)]: Chy (621:110); Lucilia (499:88); Sto (425:75); Sar (484:86); Rhi (414:74).

## Usage Notes

The dataset posted some limitations as below:

1. Annotation of the specimen until genus level. The specimen was identified until genus level due to the restriction of the morphology key provided by^7–9^, and therefore able to be reused and identified until species level, and subsequently a recognition system until species level.
2. The dataset consists of imbalanced classes of images for the genus. This was due to the removal of blurry and poor-quality images during the process of image acquisition.

## Data Availability

The original images were resized into 224 × 224 and 96 × 96 by using the web-based tools – https://teachablemachine.withgoogle.com by choosing a new image project with standard image model or embedded image model, respectively. There is no customized code in generation or processing of datasets.
